# Role of Cytokine-Inducible SH2 Domain-Containing (CISH) Protein in the Regulation of Erythropoiesis

**DOI:** 10.3390/biom13101510

**Published:** 2023-10-12

**Authors:** Saeed Maymand, Asha L. Lakkavaram, Wasan Naser, Parisa Rasighaemi, Daniel Dlugolenski, Clifford Liongue, John Stambas, Tania F. de Koning-Ward, Alister C. Ward

**Affiliations:** 1 School of Medicine, Deakin University, Waurn Ponds, Geelong, VIC 3216, Australia; saeed.maymand@education.vic.gov.au (S.M.); ashalathal@gmail.com (A.L.L.); wasan.aboud@sc.uobaghdad.edu.iq (W.N.); parisa.rasighaemi@education.vic.gov.au (P.R.); daniel.dlugolenski@10xgenomics.com (D.D.); c.liongue@deakin.edu.au (C.L.); john.stambas@deakin.edu.au (J.S.); tania.dekoning-ward@deakin.edu.au (T.F.d.K.-W.); 2 College of Science, University of Baghdad, Baghdad 10071, Iraq; 3 Institute for Mental and Physical Health and Clinical Translation, Deakin University, Waurn Ponds, Geelong, VIC 3216, Australia

**Keywords:** cytokine, CISH, EPO, SOCS

## Abstract

The cytokine-inducible SH2 domain-containing (CISH) protein was the first member of the suppressor of cytokine signaling (SOCS) family of negative feedback regulators discovered, being identified in vitro as an inducible inhibitor of erythropoietin (EPO) signaling. However, understanding of the physiological role played by CISH in erythropoiesis has remained limited. To directly assess the function of CISH in this context, mice deficient in CISH were characterized with respect to developmental, steady-state, and EPO-induced erythropoiesis. *CISH* was strongly expressed in the fetal liver, but CISH knockout (KO) mice showed only minor disruption of primitive erythropoiesis. However, adults exhibited mild macrocytic anemia coincident with subtle perturbation particularly of bone marrow erythropoiesis, with EPO-induced erythropoiesis blunted in the bone marrow of KO mice but enhanced in the spleen. *Cish* was expressed basally in the bone marrow with induction following EPO stimulation in bone marrow and spleen. Overall, this study indicates that CISH participates in the control of both basal and EPO-induced erythropoiesis in vivo.

## 1. Introduction

Erythropoietin (EPO) is a cytokine that mediates embryonic erythroid cell development and maintains appropriate adult red blood cell numbers in response to environmental signals, such as hypoxia [1]. EPO exerts its effects through its cognate homodimeric erythropoietin receptor (EPOR), with EPO binding leading to the activation of a number of intracellular pathways. These notably include the latent transcription factor Signal transducer and activator of transcription 5 (STAT5), which becomes tyrosine phosphorylated, facilitating its dimerization and nuclear translocation, where it can modulate the expression of key target genes that mediate the requisite biological effects [2]. Like other cytokine receptors, EPOR signaling is extinguished in a controlled manner by negative regulators, including suppressor of cytokine signaling (SOCS) family members [3].

The cytokine-inducible SH2 domain-containing (CISH) protein was the first member of the SOCS family discovered. It was identified in vitro as an immediate-early gene induced in hematopoietic cells by cytokines that activate STAT5, including EPO [4]. CISH binds via its SH2 domain to phosphorylated tyrosine residues of activated cytokine receptors, where it suppresses signaling via at least two mechanisms [5]. In the case of EPOR, CISH was shown to bind to the same receptor phospho-tyrosine residues as STAT5 and thereby physically block STAT5 docking [6]. It can also facilitate proteasomal degradation of activated receptor complexes through the recruitment of an E3 ubiquitin ligase complex via interactions with its SOCS box [7]. Enforced CISH expression was able to inhibit STAT5 activation and partially suppress cell proliferation mediated by EPO in vitro [4,8]. CISH was additionally able to enhance apoptosis of erythroid progenitor cells ex vivo, which correlated with the antagonism of STAT5 that protects against apoptosis [9].

Mouse studies have identified a number of functions for CISH across immunity, development, and homeostasis. Transgenic mice constitutively expressing CISH exhibited altered T and natural killer (NK) cell responses, growth retardation, and defective mammary gland development [10]. Analyses of various CISH knockout (KO) mice have revealed roles in the regulation of T cell receptor (TCR) and interleukin (IL)-4R mediated development and homeostasis of T cells [11,12], IL-15-mediated development and function of NK cells [13] and granulocyte/macrophage colony-stimulating factor receptor (GM-CSFR)-mediated myelopoiesis [14,15], as well as leptin receptor (LEPR)-mediated appetite control [16].

No erythroid phenotypes have been noted in any of the mouse studies. However, ablation of a zebrafish CISH homolog resulted in increased early erythropoiesis, including the EPOR-positive cell population [17], suggesting CISH may represent a physiological regulator of EPOR. To investigate this further, developmental, steady-state, and EPO-induced erythropoiesis was characterized in a recently described CISH KO mouse line [16]. This revealed a role for CISH in regulating in vivo erythropoiesis.

## 2. Materials and Methods

### 2.1. Mouse Studies

Previously described C57/BL6 mice harboring a mutant *Cish* allele in which the *lacZ* gene had been inserted [16] were back-crossed for 8 generations onto a Balb/c background. These *Cish*^+/−^ heterozygote (CISH HET) mice were in-crossed to yield *Cish*^+/+^ wild-type (CISH WT), CISH HET, and *Cish*^−/−^ knockout (CISH KO) progeny, with their genotype ascertained as detailed previously [16]. These mice were subsequently maintained as separate lines and fed a standard rodent chow diet ad libitum within a 12 h light/dark cycle environment. For developmental studies, timed pregnancies were used to obtain embryos at embryonic day 12.5 (E12.5) or E14.5. In other experiments, 11-week-old adult female mice were used. In some experiments, these adult mice were subjected to daily intraperitoneal injection with 100 U recombinant human EPO (Epoetin-alpha, Janssen) in saline or saline only up to 6 times. Mice were analyzed 24 h after the 4th injection (4 days), 24 h after the 6th injection (6 days), or 48 h after the 6th injection (6 + 2 days). Work was performed as approved by the Deakin University Animal Ethics Committee under the aegis of the Australian Code for the Responsible Conduct of Research. ARRIVE 2.0 Guidelines were followed with no blinding or randomization, and any animals showing signs of illness were excluded from the study.

### 2.2. Embryo Analysis

Whole embryos were fixed with 4% (*v*/*v*) paraformaldehyde (Sigma Aldrich Pty. Ltd., Castle Hill, Australia) in 0.1 M phosphate buffer (pH 7.3) (PB) (Sigma Aldrich) for 1 h and washed three times in PB. Some were stained with 100 μg/mL 5-bromo-4-chloro-3-indolyl-β-D-galactoside (X-gal) (Roche, Sigma Aldrich Pty. Ltd.) in PB supplemented with 2 mM MgCl2, 5 mM potassium ferrocyanide and 5 mM potassium ferricyanide (Sigma-Aldrich) in the dark for 12 h to detect lacZ (β-galactosidase) activity as described [18]. Embryos were imaged on a DP72 camera linked to an MVX10 microscope (Olympus Australia, Pty. Ltd., Notting Hill, Australia) using CellSens Dimension software v1.6.

### 2.3. Blood Analysis

Blood was routinely collected with minivettes (Sarstedt Australia, Mawson Lakes, Australia) and analyzed with a hematology analyzer (SCIL). Alternatively, blood was collected by cardiac puncture following euthanasia and cervical dislocation, with plasma analyzed for EPO using a Mouse EPO ELISA kit (Aviva Systems Biology, Sapphire Bioscience, Redfern, Australia).

### 2.4. Bone Marrow and Spleen Harvest

Bone marrow was extracted from the femurs and tibias by flushing with 1 mL RPMI 1640 media (Life Technologies Australia, Pty. Ltd., Mulgrave, Australia) using a 26 G needle, while whole spleens were placed in 5 mL media and passed through a 40 µm nylon mesh cell strainer (Interpath Services Pty. Ltd., Somerton, Australia). The cells were centrifuged at 1000× *g* and resuspended in an appropriate buffer to create single-cell suspensions. An aliquot was stained with trypan blue and analyzed with a Countess Automated Cell Couter (Invitrogen Australia Pty. Ltd., Mount Waverley, Australia) in order to calculate the total cell number for bone marrow and spleen.

### 2.5. FACS

To assess erythroid populations, approximately 2 × 10^6^ cells from the bone marrow or spleen were resuspended in 2% (*v*/*v*) fetal bovine serum and 0.1% (*w*/*v*) EDTA in PBS, incubated with Fc block solution (anti-CD16/CD32) before analysis with double staining using PE-conjugated anti-mouse TER-119 and APC-conjugated anti-mouse CD44 (BD Bioscience, North Ryde, Australia) as described [19], with fluorescence minus one (FMO) and unstained samples used as controls (Supplementary Figure S1). Cells were subsequently incubated with 7-AAD for 10 min prior to the acquisition of a minimum of 100,000 viable cells using a BD FACS-Canto II flow cytometer and analyzed using BD FACSDiva software (v6.0), with compensation and further characterization performed using FlowJo v.10.0.6 (BD Bioscience) software. Cell debris and background noise were removed through gating based on forward scatter (FSC)/side scatter (SSC). Single cells were gated based on the FSC area-to-height ratio, and live cells were identified by 7-AAD staining. Analysis of the TER-119+ population via CD44/FSC plots allowed quantitation of individual erythroid populations. To evaluate STAT5 phosphorylation, fixed and permeabilized cells were subjected to additional staining with AF488-conjugated anti-phospho-STAT5 (pY694) or AF488-conjugated isotype control (mouse IgG1κ). To assess proliferation, 1 × 10^5^ splenocytes were stained with CFSE and incubated in 96 well-round bottom plates containing RPMI complete cell culture medium and harvested after 24 h with CFSE staining in TER19+ cells visualized using the FlowJo V10 Proliferation Tool.

### 2.6. Colony-Forming Assays

A total of 1 × 10^5^ bone marrow or spleen cells in 1 mL methylcellulose media (R&D Systems, In Vitro Technologies, Noble Park North, Australia) were added to 35 mm tissue culture dishes (Thermo Fisher Scientific Australia Pty. Ltd., Scoresby, Australia) incubated at 37 °C with 5% CO_2_ in a humidified incubator. Manual enumeration was performed for blast-forming unit-erythroid (BFU-E) on day 8 and colony-forming unit-erythroid (CFU-E) on day 14.

### 2.7. Gene Expression Analysis

For analysis of individual genes, total RNA was extracted from approximately 2 × 10^6^ bone marrow and spleen cells using TRIsure reagent (Bioline Australia, Eveleigh, Australia) and converted to cDNA using a QuantiTect Reverse Transcription kit (Qiagen Pty. Ltd., Clayton, Australia). This was subjected to reverse-transcription PCR (RT-PCR) with primers for *Actb* (5′-TGGCATCACACCTTCTAC, 5′-AGACCATCACCAGAGTCC) and *Cish* (5′-GGACATGGTCCTTTGCGTACAG, 5′-GGAGAACGTCTTGGCTATGCAC) and samples analyzed by agarose gel electrophoresis. Quantitation was performed via area under the curve methodology using ImageJ, with expression in WT bone marrow after 4 days of EPO being set at 100%. Alternatively, for transcriptome analysis, a TruSeq Stranded Total RNA Sample Prep Kit (Illumina Australia, Melbourne, Australia) was used on bone marrow samples, and paired-end RNASeq was performed with an Illumina NovaSeq 6000 with 150 bp read lengths, which were mapped to the Ensemble GRCm38 reference genome with TopHat [20].

### 2.8. Statistical Analysis

Data were analyzed with either a Student’s *t*-test with Welch’s correction as required or a two-way analysis of variance (ANOVA)/Tukey’s multiple comparison test utilizing GraphPad Prism 8.0 with *p* < 0.05 considered significant. RNAseq gene-level count data were analyzed with DESeq2 [21], which employs a generalized linear model to identify differentially expressed genes.

## 3. Results

### 3.1. Role of CISH in Developmental Erythropoiesis

**Figure 1:**
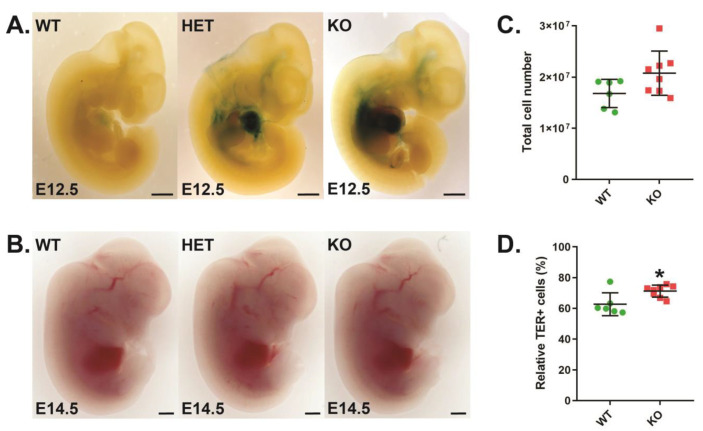
Role of CISH in fetal liver erythropoiesis. (**A**,**B**). Light microscopy of β-galactosidase staining at embryonic day 12.5 (E12.5) (**A**) and hemoglobin staining at E14.5 (**B**) in representative CISH WT, CISH HET, and CISH KO embryos as indicated, with 0.1 mm scale bars. (**C**,**D**). Quantitation of total cellularity (**C**) and relative TER119+ cells (**D**) in CISH WT (green) and CISH KO (red) E14.5 embryos. Shown are values for individual mice, along with mean ± standard error of the mean (SEM), with the level of statistical significance compared to WT indicated (* *p* < 0.05).

*Cish*^+/+^ wild-type (CISH WT), *Cish*^+/−^ heterozygote (CISH HET), and *Cish*^−/−^ knockout (CISH KO) embryos were subjected to β-galactosidase staining to detect LacZ expression from the endogenous *Cish* promoter. This revealed strong staining in the fetal liver in CISH HET and CISH KO mice that peaked at E12.5 (Figure 1A), suggesting a potential role for CISH in developmental erythropoiesis. Despite this, direct visualization revealed comparable hemoglobin pigmentation amongst all genotypes (Figure 1B), with analysis of CISH WT and CISH KO fetal livers revealing no change in overall cellularity (Figure 1C), although there was a small but significant increase in total TER119+ erythroid cells in CISH KO fetal livers (Figure 1D).

### 3.2. Role of CISH in Steady-State Erythropoiesis

Full blood examination of adult CISH KO mice revealed no significant difference in red blood cell (RBC) number or mean cell hemoglobin (MCH) compared to CISH WT mice, but subtle alterations in other red blood cell parameters were observed, including a decreased hemoglobin (Hb), hematocrit (HCT) and mean cell hemoglobin content (MCHC), but increased mean cell volume (MCV) (Table 1), consistent with mild macrocytic anemia.

**Figure 2:**
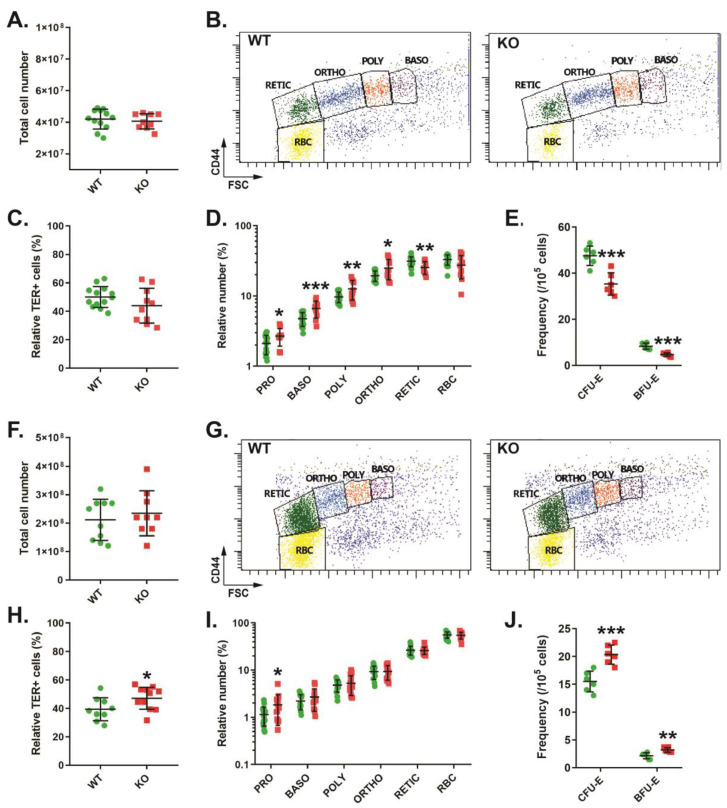
Role of CISH in steady-state adult erythropoiesis. Total cellularity (**A**,**F**), representative CD44/FSC plots of TER119+ erythroid cells (**B**,**G**), overall TER119+ erythroid cells (**C**,**H**), specific erythroid cell (**D**,**I**) and colonogenic (**E**,**J**) populations in CISH WT (green) and CISH KO (red) bone marrow (**A**–**E**) and spleen (**F**–**J**). Shown in panels (**A**,**C**–**F**,**H**–**J**) are values for individual mice, along with mean ± SEM, with statistical significance compared to WT indicated (* *p* < 0.05, ** *p* < 0.01, *** *p* < 0.001). Abbreviations: PRO: pro-erythroblast, BASO: basophilic erythroblast, POLY: polychromatic erythroblast, ORTHO: orthochromatic erythroblast, RETIC: reticulocyte, RBC: red blood cell, CFU: colony-forming unit, BFU: blast-forming unit, E: erythroid).

Analysis of bone marrow in these mice revealed no significant difference in overall cellularity (Figure 2A). Erythroid populations were analyzed by TER119/CD44 staining, as previously described [19] (Figure 2B). This indicated no change in total TER119+ erythroid cells (Figure 2C), but there was an increase in the relative proportion of pro-, basophilic, polychromatic, and orthochromatic erythroblasts and a decrease in the reticulocyte population in CISH KO mice (Figure 2D). Analysis of earlier precursors using a colony-forming assay identified decreased frequencies of both CFU-E and BFU-E (Figure 2E). The spleens of CISH KO mice were also unchanged compared to CISH WT mice with respect to cellularity (Figure 2F), but analysis with TER119/CD44 staining (Figure 2G) revealed a small but significant increase in TER119+ cells (Figure 2H). However, the only change seen in individual erythroid cell populations in the CISH KO mice was a significant increase in the pro-erythroblast population (Figure 2I), while the frequencies of both CFU-E and BFU-E precursors were also significantly increased (Figure 2J).

### 3.3. Role of CISH in EPO-Induced Erythropoiesis

EPO is a critical regulator of erythropoiesis, including stress erythropoiesis in the adult [22]. To directly investigate the potential role of CISH in regulating the in vivo functions of EPO, CISH WT and CISH KO mice were injected with EPO and erythroid parameters analyzed. In CISH WT mice, a significant increase in Hb, HCT, MCV, and MCH was observed following 4 days of EPO injection (Table 2). These parameters further increased after 6 days, by which time significant elevation of RBC counts and reduction in MCHC were also observed, with all parameters largely sustained after EPO injection was discontinued. In contrast, EPO injection of CISH KO mice led to a delayed impact on red blood cell parameters, with only MCV and MCH significantly increased by 4 days, with Hb and HCT remaining significantly decreased compared to the WT. However, after 6 days and even after EPO was discontinued, all parameters but RBC were significantly altered, with values no longer significantly different from the WT.

**Figure 3:**
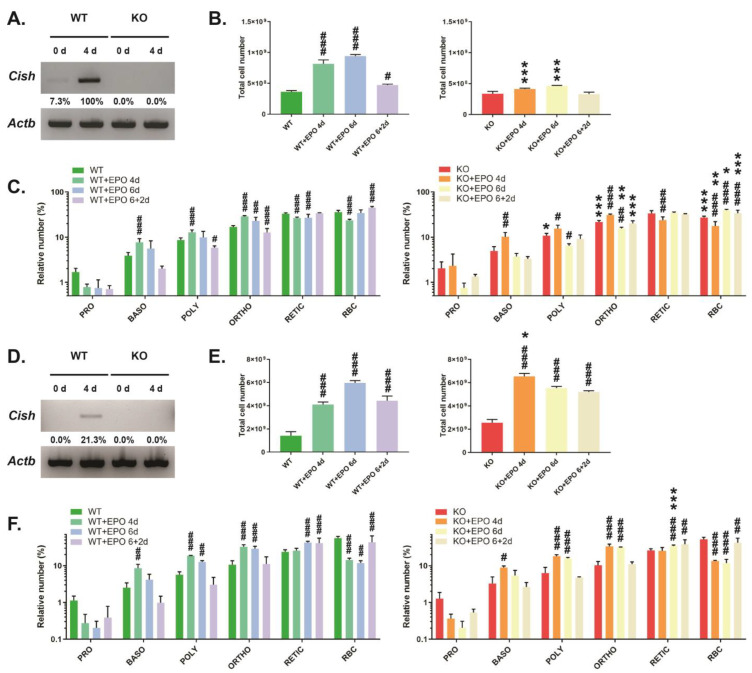
Analysis of EPO-induced erythropoiesis. Analysis of CISH WT and CISH KO mice with respect to *CISH* expression by RT-PCR (**A**,**D**), total cell number (**B**,**E**), and specific erythroid populations (**C**,**F**) in the bone marrow (**A**–**C**) and spleen (**D**–**F**). Shown in panels (**B**,**C**,**E**,**F**) are data for uninjected mice or those injected with EPO for 4 days (4d), 6 days (6d), or 6 days followed by 2 days recovery (6 + 2d). Data presented is the mean ± SEM, showing statistical significance compared to WT (* *p* < 0.05, ** *p* < 0.01, *** *p* < 0.001) or to uninjected (^#^ *p* < 0.05, ^##^ *p* < 0.01, ^###^ *p* < 0.001). (**A**,**D**) Western blot (original images can be found in Figures S4 and S5).

Analysis of the bone marrow from CISH WT mice revealed low basal *Cish* expression that was robustly increased following EPO injection, with expression absent in CISH KO mice (Figure 3A). There was a large increase in overall bone marrow cellularity in CISH WT mice following EPO stimulation, which then waned after EPO injection was stopped (Figure 3B). FACS analysis of specific erythroid populations revealed a significantly increased proportion of basophilic, polychromatic, and orthochromatic erythroblasts at 4 days, while reticulocytes and RBCs were decreased, with a gradual return toward basal proportions across the time course (Figure 3C). The EPO response was blunted in the bone marrow of CISH KO mice, which showed significantly lower total cellularity at both 4 days and 6 days (Figure 3B). The changes observed in each of the specific erythroid populations were similar to CISH WT mice, although the proportion of RBC remained reduced across the time course compared to CISH WT mice (Figure 3C).

Analysis of the spleen showed undetectable basal *Cish* expression but induction by EPO (Figure 3D). CISH WT mice demonstrated a large increase in spleen cellularity following EPO injection that declined following cessation of injection (Figure 3E). Basophilic, polychromatic, and orthochromatic erythroblasts were all significantly elevated at 4 days and slowly declined to basal levels over the time course (Figure 3F). Reticulocytes were elevated at 6 days and remained increased, while there was a significant decrease in the proportion of RBCs at 4 and 6 days that began to rebound after EPO injection was stopped. CISH KO mice showed a significantly greater initial increase in spleen cellularity at 4 days that then declined (Figure 3E). However, the changes in the relative proportions of specific erythroid cell populations in CISH KO mice largely mirrored those in CISH WT mice, with only reticulocyte percentage at 6 d showing a significant difference between genotypes (Figure 3F).

## 4. Discussion

CISH was first identified as a negative feedback regulator of EPOR signaling in vitro [4]. Subsequent studies using transgenic and knockout mice have implicated CISH in the development and function of T cells, NK cells, and myeloid cells, as well as the control of growth, mammary gland development, and appetite [10,11,12,13,14,15,16]. However, none of these studies assessed the impacts on erythropoiesis. To address this knowledge gap, this study investigated the potential physiological role of CISH in erythropoiesis through the analysis of CISH-deficient mice during primitive, steady-state end EPO-induced erythropoiesis. Collectively, these studies have identified a regulatory function for CISH in erythropoiesis.

CISH was highly expressed in the fetal liver. However, its ablation resulted in only minor perturbation of fetal erythropoiesis, reflected in an overall increase in total erythroblasts. EPOR signaling has been identified as a critical regulator of fetal erythropoiesis [1], suggesting enhanced EPOR signaling likely contributes to the phenotype observed. This is consistent with our previous study that demonstrated ablation of a CISH homolog in zebrafish resulted in increased embryonic erythropoiesis, including the EPOR-positive cell population [17].

Ablation of CISH resulted in mild macrocytic anemia in adult mice, which was confirmed in CISH KO mice on the C57/BL6 background but with no significant perturbation observed in CISH HET mice (Supplementary Table S1). Bone marrow erythropoiesis was disrupted, with a relative increase in pro-, basophilic, polychromatic, and orthochromatic erythroblasts at the expense of more mature cells, but with CFU-E and BFU-E decreased. RNAseq analysis identified a small number of genes differentially expressed in the bone marrow of CISH KO mice, the majority previously implicated in erythropoiesis or erythroid cells (Supplementary Table S2). This included decreased expression of *Mst1r*, which encodes a c-Met-related tyrosine kinase shown to mediate expansion of erythroblasts downstream of EPOR [23], *Ryk* that encodes another tyrosine kinase that facilitates Wnt5a-induced HSC quiescence [24] and *Aff4*, whose product is part of a complex that mediates the transcriptional responses of hypoxia-inducible factor (HIF)1A [25] Alternatively, increased expression was observed for *Alox15*, encoding arachidonate 15-lipoxygenase, the ablation of which leads to decreased numbers of hypochromic erythrocytes in mice [26], as well as *Rpl29* encoding the 60S ribosomal protein L29 that is highly expressed in erythroid cells [27]. There was some elevation of serum EPO, although this failed to reach statistical significance (Supplementary Figure S2). EPO-induced erythropoiesis was also blunted in the bone marrow of CISH KO mice, with the more mature populations most affected. This tissue exhibited basal *Cish* expression in CISH WT mice, which was significantly increased by EPO. Collectively, these results are consistent with in vitro studies identifying CISH as an inducible feedback regulator of EPOR signaling [4,8], but with its absence leading somewhat surprisingly to suppression of erythropoiesis within the bone marrow that correlated with perturbation of several genes implicated in this process. In contrast, CISH KO mice exhibited relatively normal splenic erythropoiesis, with EPO-induced erythropoiesis in this tissue increased, and *Cish* expression also induced in this tissue by EPO in CISH WT mice. However, EPO-stimulated splenic erythropoiesis followed a normal pattern of differentiation, with evidence of increased proliferation (Supplementary Figure S3). This is consistent with compensation and/or heightened responses in the spleen during EPO-induced erythropoiesis.

Analysis in vitro has revealed that STAT5 mediates the induction of *CISH* expression by EPO [8], facilitated via tetrameric STAT5 binding sites present in the *CISH* promoter [28], while CISH participates in direct negative regulation of EPO-induced STAT5 by competing for binding sites on the EPOR [8]. In vitro erythroid differentiation has also been shown to be influenced by the level of STAT5 activation, with differentiation maximal at higher levels, whereas intermediate levels resulted in enhanced proliferation and increased progenitors [29]. Conversely, *Stat5a*^−/−^ *Stat5b*^−/−^ mice exhibited severe microcytic anemia [30]. Together, this suggests that the EPOR/STAT5/CISH pathway is functional in vivo. Generation and characterization of lineage-specific conditional CISH KO mouse lines would provide additional insights into which cells contribute to the phenotypes observed.

## Figures and Tables

**Table 1:** Red blood cell parameters in CISH WT and KO mice.

	RBC (×10^5^/mm^3^)	Hb (g/dL)	HCT (%)	MCV (fL)	MCH (g/dL)	MCHC (%)
WT	11.3 ± 0.1	19.0 ± 0.2	55.7 ± 0.6	48.6 ± 0.1	16.8 ± 0.2	34.6 ± 0.4
KO	11.3 ± 0.2	18.0 ± 0.3 *	51.9 ± 1.4 *	49.7 ± 0.2 ***	16.7 ± 0.2	33.2 ± 0.3 *

Data is presented as mean ± SEM for CISH WT mice (WT) and CISH KO mice (KO), with statistical significance compared to WT shown (* *p* < 0.05, *** *p* < 0.001; n = 10). RBC: red blood cell count; Hb: hemoglobin; HCT: hematocrit; MCV: mean cell volume; MCH: mean cell hemoglobin; MCHC: mean cell hemoglobin concentration.

**Table 2:** Red blood cell parameters in CISH WT and KO mice injected with EPO.

	RBC (×10^5^/mm^3^)	Hb (g/dL)	HCT (%)	MCV (fL)	MCH (g/dL)	MCHC (%)
WT	11.0 ± 0.1	18.4 ± 0.2	55.1 ± 0.5	48.8 ± 0.2	16.5 ± 0.2	34.4 ± 0.5
WT + EPO 4d	11.9 ± 0.4	20.1 ± 0.7 ^#^	59.5 ± 1.5 ^#^	51.3 ± 0.6 ^##^	17.2 ± 0.2 ^#^	33.1 ± 0.4
WT +EPO 6d	12.8 ± 0.5 ^##^	21.9 ± 2.2 ^###^	68.0 ± 1.5 ^##^	55.7 ± 0.2 ^###^	17.7 ± 0.3 ^#^	31.7 ± 0.6 ^#^
WT + EPO 6 + 2d	12.9 ± 0.6 ^##^	21.6 ± 0.8 ^###^	66.2 ± 2.0 ^##^	54.3 ± 1.2 ^###^	17.4 ± 0.3 ^#^	31.7 ± 0.2 ^#^
KO	11.6 ± 0.6	16.7 ± 0.5 **	47.8 ± 2.4 *	49.5 ± 0.2 *	16.7 ± 0.1	33.0 ± 0.2 *
KO + EPO 4d	10.5 ± 0.8	17.1 ± 0.8 *	49.4 ± 2.4 *	52.5 ± 1.0 ^##^	17.4 ± 0.3 ^#^	33.6 ± 0.3
KO + EPO 6d	12.3 ± 0.9	19.7 ± 0.7 ^#^	60.8 ± 2.6 ^#^	55.0 ± 1.0 ^###^	18.2 ± 0.7 ^#^	31.9 ± 0.2 ^#^
KO + EPO 6 + 2d	13.0 ± 0.9	20.4 ± 0.8 ^#^	66.0 ± 2.7 ^##^	54.0 ± 0.6 ^###^	17.2 ± 0.1 ^#^	31.9 ± 0.3 ^##^

Data is presented as mean ± SEM for CISH WT (WT) and CISH KO (KO) mice, with statistical significance shown in comparison to WT (* *p* < 0.05, ** *p* < 0.01) or to uninjected (^#^ *p* < 0.05, ^##^ *p* < 0.01, ^###^ *p* < 0.001).

## Data Availability

All data analyzed during this study are included in the published article (and associated Supplementary Materials) or are available by request.

## Supplementary Materials

The following supporting information can be downloaded at https://www.mdpi.com/article/10.3390/biom13101510/s1.
