# Supplementary material for: Role of Cytokine-Inducible SH2 Domain-Containing (CISH) Protein in the Regulation of Erythropoiesis
Source: Biomolecules. 2023 Oct 12;13(10):1510. doi: 10.3390/biom13101510 (PMC10604548; doi:10.3390/biom13101510)
Supplement: Supplementary file 1 [file biomolecules-13-01510-s001.zip › biomolecules-2619612-supplementary.pdf]

Supp. Table S1. Red blood cell parameters in Cish WT, HET and KO C57/BL6 mice.

	**RBC ($\times 10^5$/mm^3)**	**Hb (g/dL)**	**HCT (%)**	**MCV (fL)**	**MCH (g/dL)**
WT	10.2 ± 0.6	16.4 ± 0.4	48.7 ± 0.8	49.0 ± 0.4	16.4 ± 0.4
HET	9.9 ± 0.3	16.2 ± 0.6	49.2 ± 1.1	49.3 ± 0.8	16.2 ± 0.2
KO	9.6 ± 0.3	15.1 ± 0.3*	45.0 ± 0.7*	50.5 ± 0.3*	16.8 ± 0.3

Data is presented as mean ± SEM for Cish WT, HET and KO mice, with statistical significance compared to WT shown (* $p<0.05$; n=4). RBC: red blood cell count; Hb: hemoglobin; HCT: hematocrit; MCV: mean cell volume; MCH: mean cell hemoglobin.

Supp. Table S2. Differentially expressed genes in Cish KO versus WT bone marrow.

Gene name	Gene description	ID	logFC	FDR
Gm13841	pseudogene	ENSMUSG00000083833	-12.77634886	1.91E-59
Gm8210	pseudogene	ENSMUSG00000094497	-11.39177581	1.91E-59
Mst1r	macrophage stimulating 1 receptor	ENSMUSG00000032584	-9.38949571	3.84E-06
Aff4	AF4/FMR2 family, member 4	ENSMUSG00000049470	-3.222573225	0.030208062
Ryk	receptor-like tyrosine kinase	ENSMUSG00000032547	-2.536816371	2.72E-07
Alox15	arachidonate 15-lipoxygenase	ENSMUSG00000018924	2.130038845	0.030208062
Rpl29	ribosomal protein L29	ENSMUSG00000048758	3.369245988	2.97E-34

Genes with an FDR p<0.05 from a comparison of Cish WT and KO mice (n=6) are shown. ID: identification number; FC: fold-change; FDR: false discovery rate.

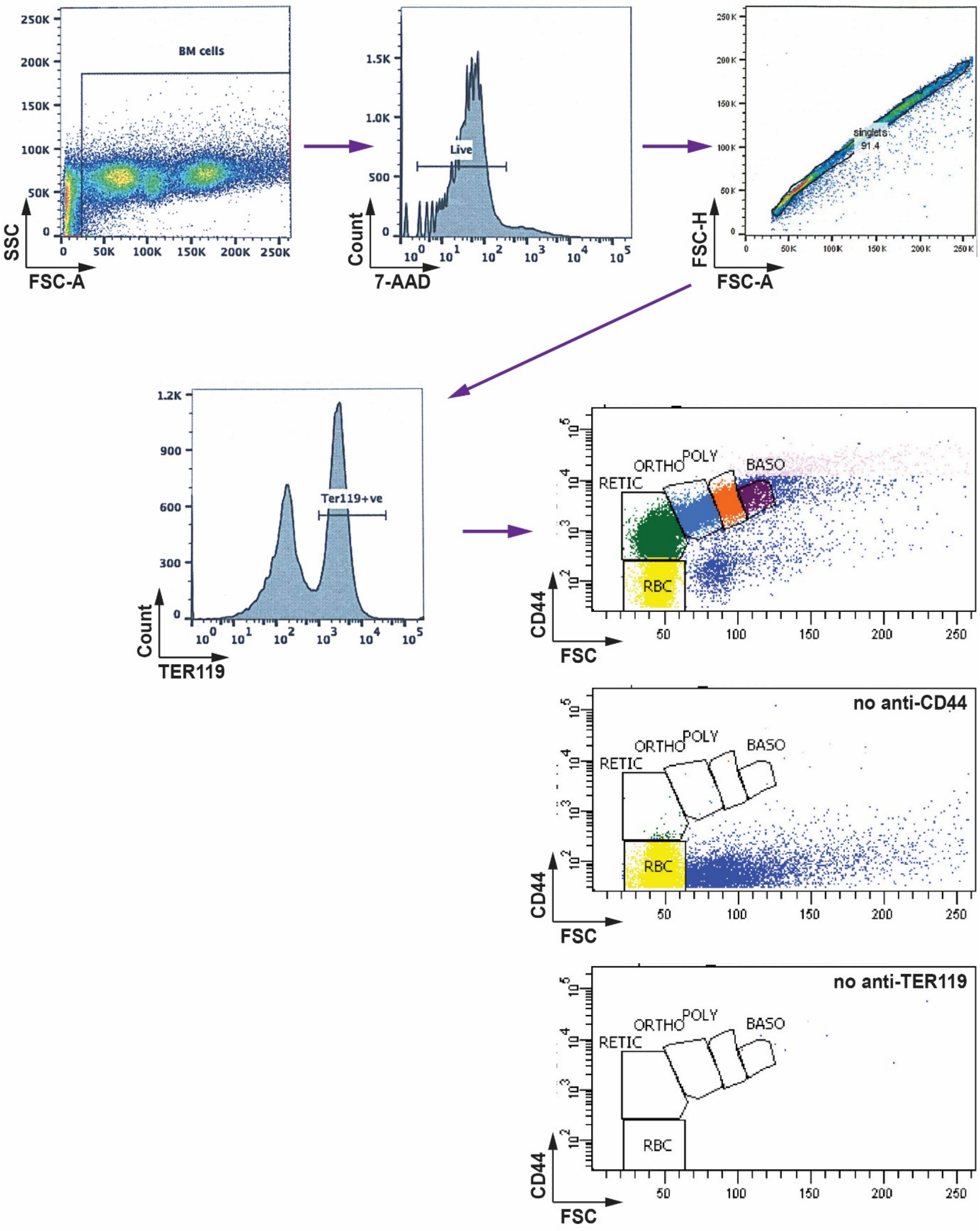

Supp. Figure S1. Flow cytometry strategy and controls. Strategy to quantify erythroid cell populations, showing representative plots from bone marrow samples from a Cish WT Balb/c mouse. Total cells were gated on the basis of morphology on a side scatter area (SSC) versus forward scatter area (FSC-A) plot, with viable cells gated on 7-AAD staining and single cells gated on a forward scatter height (FSC-H) versus FSC-A plot,. TER119+ cells were then gated, with individual erythroid cell populations identified by gating on a CD44/FSC plot of these cells as indicated, with FMO controls, no anti-CD4 and no anti-TER119, shown below.

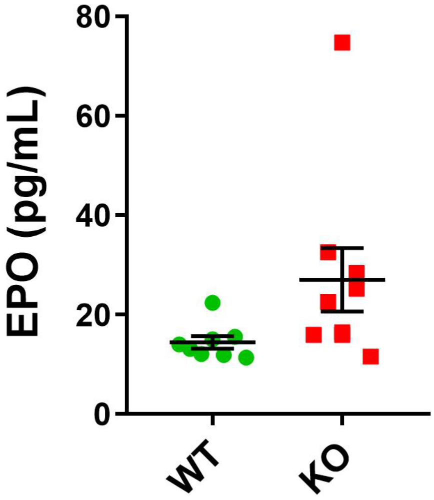

Supp. Figure S2. Analysis of EPO levels. Quantitation of plasma EPO concentration in Cish WT and KO mice. Shown are values for individual mice, along with mean ± standard error of the mean (SEM). No statistical difference between WT and KO was observed.

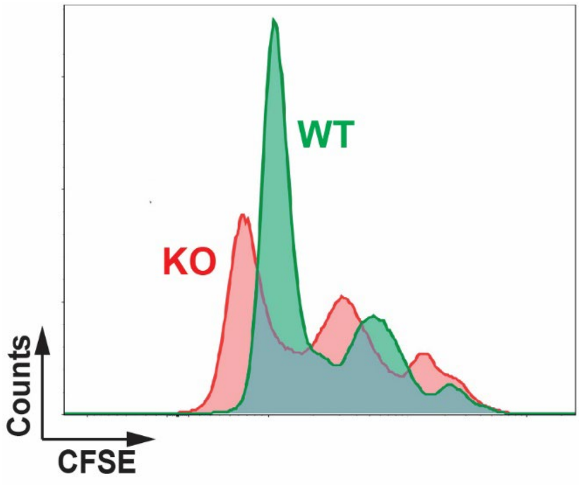

Supp. Figure S3. Proliferation of spleen erythroid cells. Analysis of CFSE staining as a measure of proliferation in TER119+ spleen cells from representative EPO-treated Cish WT and Cish KO mice.
