# Supplementary figures and images for: Role of Cytokine-Inducible SH2 Domain-Containing (CISH) Protein in the Regulation of Erythropoiesis
Source: Biomolecules. 2023 Oct 12;13(10):1510. doi: 10.3390/biom13101510 (PMC10604548; doi:10.3390/biom13101510)

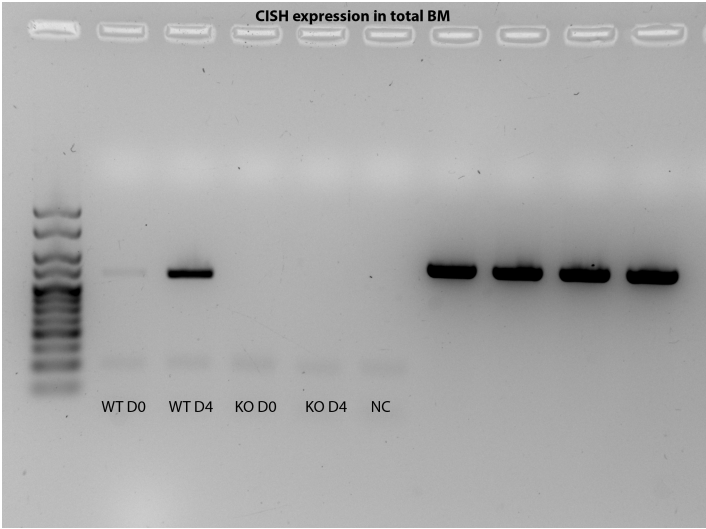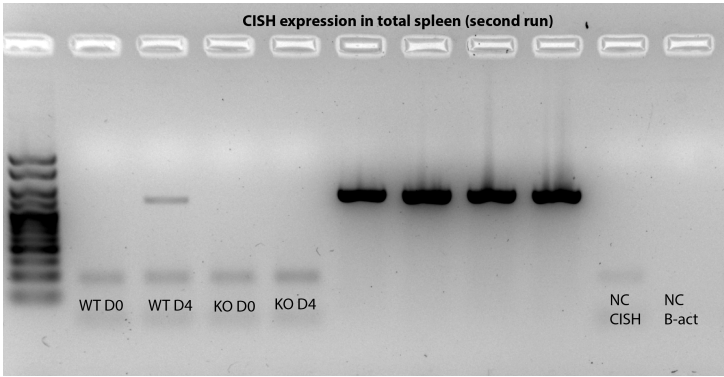

Supplement: Supplementary file 1 [file biomolecules-13-01510-s001.zip › Figure S4 and Figure S5.pdf]
